# Manipulating regulatory T cells: is it the key to unlocking effective immunotherapy for pancreatic ductal adenocarcinoma?

**DOI:** 10.3389/fimmu.2024.1406250

**Published:** 2024-05-30

**Authors:** Henry Smith, Edward Arbe-Barnes, Enas Abu Shah, Shivan Sivakumar

**Affiliations:** ^1^ School of Medicine and Biomedical Sciences, University of Oxford, Oxford, United Kingdom; ^2^ Institute of Immunology and Transplantation, University College London, London, United Kingdom; ^3^ Sir William Dunn School of Pathology, University of Oxford, Oxford, United Kingdom; ^4^ Institute of Immunology and Immunotherapy, Birmingham Medical School, Birmingham, United Kingdom; ^5^ Birmingham Cancer Centre, Queen Elizabeth Hospital, Birmingham, United Kingdom

**Keywords:** immunotherapy, regulatory T cells, pancreatic ductal adenocarcinoma, TIGIT, CCR8, Helios, adenosine

## Abstract

The five-year survival rates for pancreatic ductal adenocarcinoma (PDAC) have scarcely improved over the last half-century. It is inherently resistant to FDA-approved immunotherapies, which have transformed the outlook for patients with other advanced solid tumours. Accumulating evidence relates this resistance to its hallmark immunosuppressive milieu, which instils progressive dysfunction among tumour-infiltrating effector T cells. This milieu is established at the inception of neoplasia by immunosuppressive cellular populations, including regulatory T cells (T_regs_), which accumulate in parallel with the progression to malignant PDAC. Thus, the therapeutic manipulation of T_regs_ has captured significant scientific and commercial attention, bolstered by the discovery that an abundance of tumour-infiltrating T_regs_ correlates with a poor prognosis in PDAC patients. Herein, we propose a mechanism for the resistance of PDAC to anti-PD-1 and CTLA-4 immunotherapies and re-assess the rationale for pursuing T_reg_-targeted therapies in light of recent studies that profiled the immune landscape of patient-derived tumour samples. We evaluate strategies that are emerging to limit T_reg_-mediated immunosuppression for the treatment of PDAC, and signpost early-stage trials that provide preliminary evidence of clinical activity. In this context, we find a compelling argument for investment in the ongoing development of T_reg_-targeted immunotherapies for PDAC.

## 1 Introduction

Since 1863 – when Rudolf Virchow first observed leukocyte infiltrates decorating neoplastic tissues – research has uncovered a dynamic interplay between the immune system and pre-malignant cells, which governs their progressive transformation to invasive derivatives (1). In parallel, efforts to leverage the immune system to treat malignancy have a long history; in 1868, Wilhelm Busch reported tumour regression after intentionally infecting patients with *Streptococcus pyogenes (* 2). Today, immunotherapy has revolutionised clinical oncology: immune checkpoint inhibitors (ICIs; specifically anti-PD-1, -CTLA-4, and -PD-L1 antibodies) provide unprecedented rates of durable anti-tumour responses in patients with several types of cancer (3). However, ICIs, including the combination of anti-CTLA-4 and anti-PD-L1 antibodies, have yielded limited responses in pancreatic ductal adenocarcinoma (PDAC); a malignancy of the exocrine pancreas that constitutes 95% of pancreatic cancer cases (4, 5). Accordingly, PDAC carries a bleak prognosis: globally, the 5-year survival rate at the time of diagnosis is 9% (6).

Substantial research has sought to identify immunological mechanisms that render PDAC resistant to ICIs. Concomitantly, these studies have unearthed therapeutic targets that could feasibly be exploited to induce anti-tumour immunity in PDAC; indeed, strategies to restrain immunosuppressive regulatory T cells (T_regs_), myeloid cells, and cancer-associated fibroblasts are currently under development (7, 8). The manipulation of CD4^+^ T_regs_ has gained considerable traction, stemming from the discovery that an abundance of intratumoral T_regs_ correlates with a poor prognosis in PDAC patients (9). Herein, we propose a mechanism for the intrinsic resistance of PDAC to ICIs; discuss the rationale for pursuing T_reg_-targeted therapies in the context of PDAC; and evaluate emerging strategies to limit T_reg_-mediated immunosuppression. Overall, we argue that T_reg_-targeted immunotherapies offer a valuable opportunity to improve clinical outcomes in PDAC.

## 2 Why have ICIs proved ineffective in the context of PDAC?

Any effective immunotherapy must induce lasting anti-tumour immunity, typically mediated by CD4^+^ and CD8^+^ effector T (T_eff_) cells and directed against tumour-associated antigens acquired during malignant progression (10, 11). Researchers have sought to identify immunological mechanisms that render PDAC resistant to ICIs. Initial efforts utilised autochthonous murine models of PDAC: *Kras* ^LSL-G12D/+^;*Pdx-1-Cre* (KC) and *Kras* ^LSL-G12D/+^;*Trp53* ^LSL-R172H/+^;*Pdx-1-Cre* (KPC), which recapitulate features of the human disease (12, 13). More recent analyses have profiled the immune landscape of patient-derived tumour samples, facilitated by advances in single-cell multi-omic technologies (14–16).

It is well established that the baseline density of tumour-infiltrating T_eff_ cells is a critical determinant of therapeutic responses to ICIs (17, 18). Thus, the immunologically ‘cold’ phenotype that characterises PDAC has often been attributed to the physical exclusion of T_eff_ cells from the tumour microenvironment (TME) (19, 20). However, recent studies have challenged this paradigm, identifying heterogenous baseline infiltrates of CD4^+^ and CD8^+^ T_eff_ cells that correlate with prolonged overall survival in PDAC patients (14, 15, 21–26). There is also evidence for ongoing anti-tumour immunity; Freed-Pastor et al. identified a population of HLA-DR^+^Ki67^+^CD57^-^CD8^+^ T cells – indicative of an activated, proliferative phenotype – that are present in the majority of patients (27). Altogether, these studies suggest that inducing T_eff_ cell-mediated anti-tumour immunity in PDAC may not be as intractable as is widely considered (23).

In further support of this notion, a rare subset (~1.6.%) of PDAC patients with hypermutated mismatch repair deficient (dMMR) tumours exhibit marked therapeutic responses to anti-PD-1 antibodies (28). These tumours present a broad repertoire of neoantigens, which direct potent anti-tumour immune responses (29, 30). Indeed, in this patient cohort, sequencing of the TCR Vβ chain revealed that 94% of intratumoral T cell clonotypes were unique to tumours, implying the existence of a neoantigen-specific immune response (24). Overall, this highlights the importance of neoantigens as a substrate for T_eff_-mediated anti-tumour immunity – indeed, on the basis of this principle, pembrolizumab and nivolumab (anti-PD-1) were granted FDA-approval in 2017 for the treatment of dMMR tumours, irrespective of their tissue of origin (31). In this context, it is notable that recent studies have challenged the claim that MMR-proficient PDAC harbours a limited repertoire of neoantigens. Freed-Pastor et al. investigated a cohort of 57 advanced PDAC patients and discovered that they all possessed neoepitopes with predicted ability to bind MHC class-I molecules (27). Accordingly, studies have consistently identified intratumoral neoantigen-reactive CD8^+^ T cells in PDAC patients, indicating that these neoantigens are capable of directing anti-tumour immunity (27, 32, 33).

Nevertheless, it is evident that this population of intratumoral neoantigen-reactive CD8^+^ T cells is not sufficient to drive therapeutic responses to FDA-approved ICIs in MMR-proficient PDAC. Indeed, multi-omic profiling of the PDAC immune landscape in resectable patients has revealed that ‘dysfunctional’ and ‘senescent’ phenotypes – both hypofunctional states, defined by the expression of multiple inhibitory receptors: TIGIT, LAG-3, TIM-3, and CD39 – dominate the intratumoral T_eff_ cell repertoire, leaving few activated T cells that are thus unable to control the tumour (15, 25). In addition, a more pronounced exhaustion signature has been observed in CD8^+^ T cells from fine-needle biopsy samples of advanced, unresectable PDAC (14).

This progressive dysfunction of intratumoral T_eff_ cells can be attributed to the profoundly immunosuppressive TME. It is established by the progressive infiltration of immunosuppressive cells: T_regs_, myeloid-derived suppressor cells, neutrophils, and tumour-associated macrophages (34). In the murine KC model, these populations dominate the immune landscape of pancreatic intraepithelial neoplasia (PanIN): precursor lesions that culminate in the development of PDAC (19). Other non-immune cellular populations also contribute to the immunosuppressive TME. For example, a subset of cancer-associated fibroblasts present antigenic peptides in association with MHC class-II molecules; however, they lack expression of classical co-stimulatory molecules and thus command CD4^+^ T cells to the T_reg_ lineage (35). In summary, neoantigen-specific T_eff_ responses are dampened by the gradual accumulation of immunosuppressive cells in the TME, which dictates the progression from PanIN to PDAC. Hence, the development of immunomodulatory therapies for PDAC must focus on surmounting the hallmark immunosuppressive TME (36). Importantly, the progressive nature of intratumoral T_eff_ cell dysfunction promises to confer a broad window during which such therapies might be effective.

## 3 What is the phenotype of T_regs_ in PDAC?

To date, strategies targeting myeloid-derived suppressor cells or cancer-associated fibroblasts for the treatment of PDAC have generally failed to demonstrate therapeutic promise in clinical trials (37–40). However, one promising strategy – which has gained substantial traction in the context of PDAC – is combatting T_reg_-mediated immunosuppression. This originated from the discovery that an abundance of intratumoral T_regs_ correlates with a poorer prognosis in PDAC patients (9). Accordingly, the depletion of T_regs_ has been shown to delay tumour growth in orthotopically transplanted murine PDAC, albeit with conflicting results from other murine models (41, 42). However, recent single-cell analyses have uncovered extensive diversity among intratumoral T_regs_; in this context, it is important to re-evaluate the rationale for the development of T_reg_-targeted therapies.

### 3.1 Effector T_regs_ are highly immunosuppressive

Classically, CD4^+^ T_regs_ have been defined according to expression of FOXP3, considered a lineage-specifying transcription factor (TF), or the interleukin (IL)-2 receptor α chain (CD25). In a seminal study, Hiraoka et al. discovered that the prevalence of FOXP3^+^ T_regs_ increases during the progression from PanIN to advanced PDAC – at this latter stage, they constitute 35% (± 11%) of the total intratumoral CD4^+^ population (9, 15). Further, it is estimated that 54% (± 19%) of intratumoral T_regs_ are effector T_regs_ (eT_regs_; CD45RA^-^FOXP3^hi^CD25^hi^) (15). These cells express high levels of TIGIT, CTLA-4, ICOS, CD39, and HLA-DR, which are indicative of functional activation and potent immunosuppressive capacity (14, 15). This activated state has been attributed to sustained TCR stimulation, provided by the plethora of self- and quasi-self-antigens present in the inflammatory TME (43). However, a stable eT_reg_ phenotype is also dependent on the expression of Helios, a member of the Ikaros TF family. Indeed, intratumoral Helios^+^ T_regs_ exhibit significantly higher expression of FOXP3, compared to Helios^-^ T_regs_ (44).

Intratumoral eT_regs_ potently suppress CD8^+^ T cell-mediated immunity via the expression of co-inhibitory molecules e.g., CTLA-4, which prevents the functional maturation of dendritic cells (41); secretion of immunosuppressive cytokines e.g., IL-10, IL-35, and TGF-*β*; sequestration of IL-2, which hampers IL-2-dependent T cell activation; and the secretion of granzymes to lyse target CD8^+^ cells (45). In support of their immunosuppressive capacity *in situ*, spatial analyses reveal that 90% of T_regs_ reside in close proximity to a CD8^+^ T cell in the PDAC TME (15).

### 3.2 FOXP3^+^RORγt^+^ T_regs_ provide mitogenic signalling

FOXP3^+^ T_regs_ exhibit extensive heterogeneity in PDAC. Strikingly, studies have discovered populations of FOXP3^+^ T_regs_ that, in addition to IL-10, secrete high levels of pro-inflammatory cytokines. For example, Chellappa et al. identified T_regs_ that co-express FOXP3 and RORγt: a factor that specifies the type-17 T-helper cell lineage (T_H_17) (46). These cells retained markers associated with FOXP3^+^ T_regs_ e.g., CTLA-4, CD39, and ICOS, indicating an ability to robustly suppress anti-tumour immunity. However, through the simultaneous production of IL-17, these FOXP3^+^RORγt^+^ cells provide mitogenic signalling to transformed pancreatic epithelial cells, which upregulate the IL-17 receptor (47). Moreover, studies have identified populations of FOXP3^-^ T_reg_-like cells that share expression of molecules classically associated with immunosuppressive T_reg_ functions (e.g., IL-10, CCR8, TIGIT, ICOS, CTLA-4) (48, 49). Barilla et al. demonstrated that the gene expression profile of one such population, termed T_r_1 cells (*CD49b*, *CD73*, and *AHR*), was associated with decreased overall survival in PDAC patients (49). Furthermore, Whiteside et al. suggest that intratumoral T_eff_ cells may adopt this FOXP3^-^ T_reg_-like phenotype following the ablation of FOXP3^+^ cells (48).

**Figure 1:**
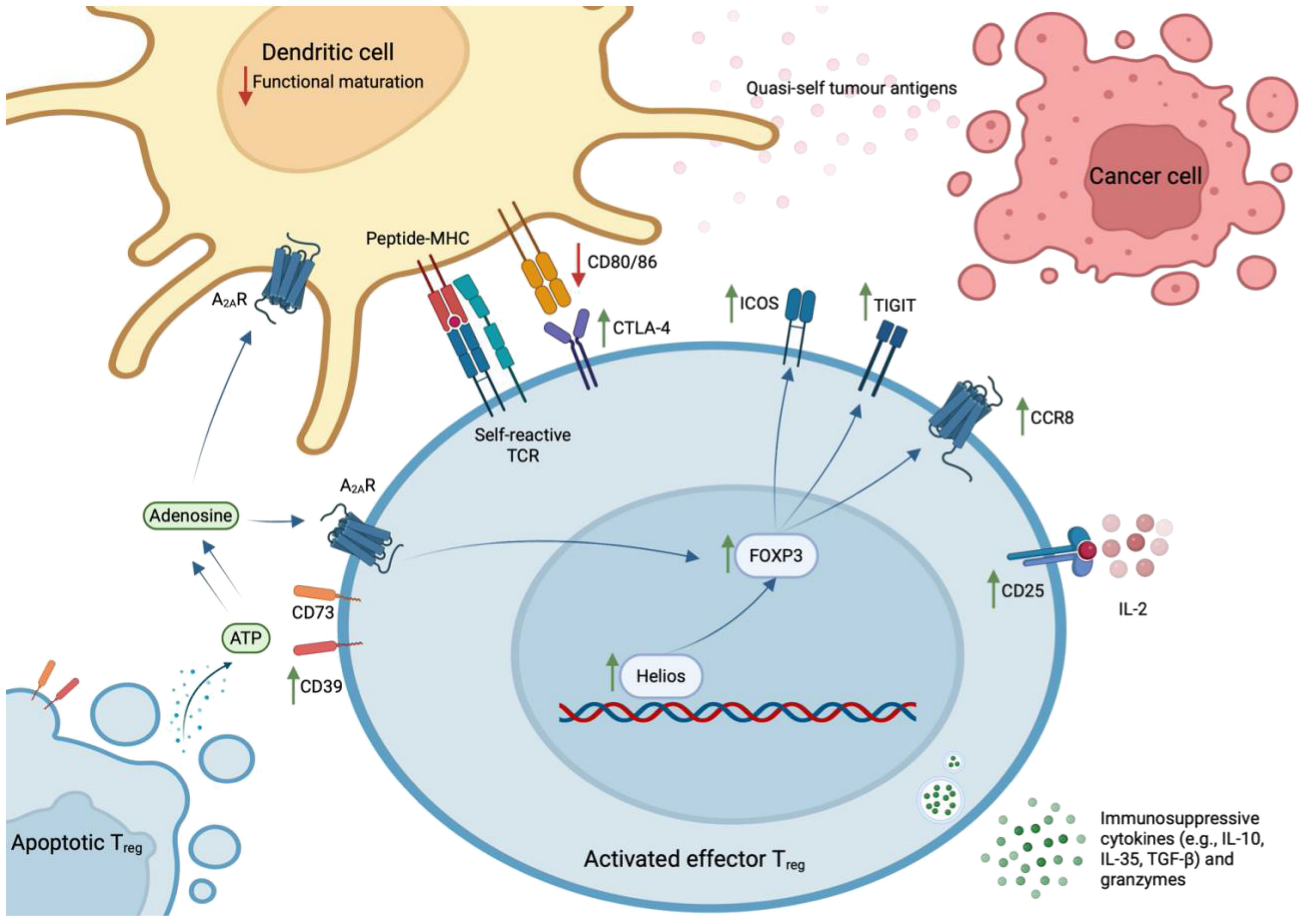
Phenotype of effector T_reg_ cells in human PDAC. Effector T_regs_ – characterised by the expression of FOXP3, CD25, TIGIT, CTLA-4, ICOS, CD39, and CCR8 – are activated by sustained TCR stimulation with abundant self- and quasi-self-antigens and stabilised by expression of the Helios transcription factor. These cells exhibit potent immunosuppressive capacity within the PDAC TME, where they exist in close proximity to CD8^+^ T lymphocytes. Specifically, they express co-inhibitory molecules (e.g., CTLA-4, TIGIT, ICOS); convert ATP to immunosuppressive adenosine via ectoenzymes that remain catalytically active after cell-death (CD39 and CD73); secrete immunosuppressive cytokines (e.g., IL-10, IL-35, TGF-*β*) and granzymes that lyse CD8^+^ T_eff_ cells; and sequester IL-2 that is required for T_eff_ cell activation.

This profound heterogeneity likely explains conflicting reports regarding the overall contribution of T_regs_ in the pathophysiology of PDAC. One notable study reported an increased prevalence of T_regs_ in tumours of long-term PDAC survivors (24). Moreover, depletion of T_regs_ prior to the development of PanIN in KC mice has been shown to accelerate malignant progression (42). Conceivably, the use of different experimental systems, including varied methods for detecting and defining intratumoral T_regs_, might accentuate specific T_reg_-associated functions and thereby explain these conflicting reports. Moreover, studies have suggested that, as part of normal immune homeostasis, intratumoral T_regs_ accompany CD8^+^ T cell infiltrates (21, 42, 49), which may further obscure any relationship between the prevalence of intratumoral T_regs_ and a poor prognosis. Nevertheless, harnessing the therapeutic manipulation of T_regs_ will require a targeted approach, based on a detailed understanding of the heterogeneous functions ascribed to T_regs_, and their spatiotemporal dynamics in the PDAC TME (**Figure 1**). In addition, such an approach will reduce the systemic side-effects associated with T_reg_-targeted immunotherapies.

### 3.3 Apoptotic T_regs_ are paradoxically immunosuppressive

This hypothesis is fortified by the discovery that apoptotic T_regs_, defined by increased expression of Ki67 and cleaved caspase-3, exert immunosuppressive effects in the oxidative TME. They release large quantities of ATP, which is converted into adenosine via CD39 and CD73 – ectoenzymes that are expressed by T_regs_ and remain catalytically active after cell-death (50). Through the A_2A_ receptor, accumulating extracellular adenosine inhibits T_eff_ cell proliferation; induces immunosuppressive dendritic cells; and stabilises surviving T_regs_ (51). Thus, CD39 and CD73 expression correlate with a poor prognosis in patients with various solid tumours (52, 53). Importantly, this paracrine signalling pathway is likely to be operating in human PDAC, as intratumoral T_regs_ express high levels of CD39.

## 4 What are the strategies to manipulate T_regs_ for the treatment of PDAC?

The manipulation of T_regs_ has captured significant attention from both scientific and commercial communities as a novel approach to the treatment of PDAC. The earliest attempts depleted T_regs_ by targeting CD25 with antibodies, daclizumab, or the IL-2-diphtheria toxin fusion protein, ONTAK (54, 55). However, IL-2 signalling via CD25 promotes the survival of activated T_eff_ cells, conferring a limited therapeutic window to CD25-targeted interventions. Nevertheless, these efforts provided proof-of-concept for the therapeutic manipulation of T_regs_. Today, numerous T_reg_-targeted therapies are under development for the treatment of advanced solid tumours, including PDAC (**Table 1**).

**Table 1:** T_reg_-targeted immunotherapies in current development (as of 01/05/2024).

Drug	Sponsor	Properties	Status	Reference
CTLA-4				
Botensilimab (AGEN1181)	Agenus Inc.	Fc-engineered anti-CTLA-4 monoclonal antibody	Phase I/II, in combination with gemcitabine and nab-paclitaxel, in patients with metastatic PDAC	NCT05630183
ONC-392	OncoC4	Fc-engineered anti-CTLA-4 monoclonal antibody	Phase I/II, +/- pembrolizumab (anti-PD-1), in patients with advanced solid tumours, including PDAC	NCT04140526
XTX101	Xilio Therapeutics	Fc-engineered anti-CTLA-4 monoclonal antibody	Phase I/II, +/- atezolizumab (anti-PD-L1), in patients with advanced solid tumours	NCT04896697
TIGIT				
Tiragolumab (MTIG7192A)	Roche/Genentech	Anti-TIGIT monoclonal antibody	Phase I/II, in combination with atezolizumab (anti-PD-L1) and chemotherapy, in patients with metastatic PDAC	NCT03193190
Domvanalimab (AB154)	Arcus Biosciences/Gilead Sciences	Fc-silent anti-TIGIT monoclonal antibody	Phase I/II trial, in combination with zimberelimab and APX005M (agonistic CD40), in patients with metastatic PDAC	NCT05419479
AB308	Arcus Biosciences/Gilead Sciences	Fc-enabled anti-TIGIT monoclonal antibody	Phase Ib, in combination with zimberelimab (anti-PD-1), in patients with advanced solid tumours	NCT04772989
Vibostolimab (MK-7684)	Merck Sharp & Dohme	Anti-TIGIT monoclonal antibody	Phase I, +/- pembrolizumab (anti-PD-1) +/- chemotherapy, in patients with advanced solid tumours	NCT02964013
Belrestotug (EOS-448)	GlaxoSmithKline/iTeos Therapeutics	Anti-TIGIT monoclonal antibody	Phase I/II, +/- pembrolizumab or dostarlimab (anti-PD-1) +/- inupadenant (selective A2aR antagonist) +/- chemotherapy, in patients with advanced solid tumours	NCT05060432
Ociperlimab (BGB-A1217)	BeiGene	Anti-TIGIT monoclonal antibody	Phase I, +/- tislelizumab (anti-PD-1) +/- chemotherapy, in patients with advanced solid tumours	NCT04047862
PM1021	Biotheus	Anti-TIGIT monoclonal antibody	Phase I, +/- PM8001 (anti-PDL1-TGFb), in patients with advanced solid tumours	NCT05537051
Etigilimab (MPH313)	Mereo BioPharma	Anti-TIGIT monoclonal antibody	Phase I/II, in combination with nivolumab (anti-PD-1), in patients with advanced solid tumours	NCT04761198
BAT6005	Bio-Thera Solutions	Anti-TIGIT monoclonal antibody	Phase I in patients with advanced or metastatic solid tumours	NCT05116709
HB0030	Shanghai Huaota Pharmaceuticals	Anti-TIGIT monoclonal antibody	Phase I in patients with advanced solid tumours	NCT05706207
JS006	Shanghai Junshi Biosciences	Anti-TIGIT monoclonal antibody	Phase I, +/- toripalimab (anti-PD-1), in patients with advanced solid tumours	NCT05061628
AK127	Akeso	Anti-TIGIT monoclonal antibody	Phase I/II, in combination with AK104 (anti-CTLA4-PD1 bispecific), in patients with advanced or metastatic solid tumours	NCT05021120
COM902	Compugen	Anti-TIGIT monoclonal antibody	Phase I, +/- COM701 (anti-PVRIG*), in patients with advanced solid tumours	NCT04354246
CHS-006	Coherus BioSciences	Anti-TIGIT monoclonal antibody	Phase I, in combination with toripalimab (anti-PD-1), in patients with advanced solid tumours	NCT05757492
BMS-986442	Bristol Myers Squibb/Agenus Inc.	Anti-TIGIT bispecific antibody (other target is undisclosed)	Phase I/II, in combination with nivolumab (anti-PD-1) +/- chemotherapy, in patients with advanced solid tumours	NCT05543629
HB0036	Shanghai Huaota Pharmaceuticals	Anti-TIGIT-PDL1 bispecific antibody	Phase I/II in patients with advanced solid tumours	NCT05417321
PM1022	Biotheus	Anti-TIGIT-PDL1 bispecific antibody	Phase I/II in patients with advanced solid tumours	NCT05867771
PM1009	Biotheus	Anti-TIGIT-PVRIG bispecific antibody	Phase I in patients with advanced solid tumours	NCT05607563
HLX301	Shanghai Henlius Biotech	Anti-TIGIT-PDL1 bispecific antibody	Phase I/II in patients with advanced or metastatic solid tumours	NCT05102214
HLX53	Shanghai Henlius Biotech	Anti-TIGIT Fc fusion protein	Phase I in patients with advanced solid tumours	NCT05394168
ICOS				
Alomfilimab (KY1044)	Kymab/Sanofi	Agonistic ICOS monoclonal antibody	Phase I/II, +/- atezolizumab (anti-PD-L1), in patients with advanced solid tumours including PDAC	NCT03829501
Vopratelimab (JTX-2011)	Jounce Therapeutics	Agonistic ICOS monoclonal antibody	Phase II, in combination with pimivalimab (anti-PD-1), in patients with non-small cell lung cancer	NCT04549025
Helios				
DKY709	Novartis Pharmaceuticals	Selective Helios degrader	Phase I, +/- PDR001 (anti-PD-1), in patients with advanced solid tumours	NCT03891953
PLX-4545	Plexium	Selective Helios degrader	Pre-clinical development	https://www.plexium.com/therapeutic-areas-plexium-e3-ligase-drugs/
Helios CELMoD	Bristol Myers Squibb	Selective Helios degrader	Pre-clinical development	https://www.bms.com/researchers-and-partners/in-the-pipeline.html
CD25				
Vopikitug (RG6292)	Roche/Genentech	Anti-CD25 monoclonal antibody	Phase I, +/- atezolizumab (anti-PD-L1), in patients with advanced solid tumours	NCT04158583
AU-007	Aulos Bioscience Inc.	Anti-IL-2 monoclonal antibody	Phase I/II, +/- aldesleukin (recombinant IL-2), in patients with locally advanced or metastatic solid tumours	NCT05267626
CCR8				
BMS-986340	Bristol Myers Squibb	Non-fucosylated anti-CCR8 monoclonal antibody	Phase I/II, +/- nivolumab (anti-PD-1) +/- docetaxel, in patients with advanced solid tumours including PDAC	NCT04895709
CHS-114	Coherus BioSciences	Afucosylated anti-CCR8 monoclonal antibody	Phase I/II in patients with advanced solid tumours	NCT05635643
BAY3375968	Bayer	Afucosylated anti-CCR8 monoclonal antibody	Phase I, +/- pembrolizumab (anti-PD-1), in patients with advanced solid tumours	NCT05537740
GS-1811	Gilead Sciences	Afucosylated anti-CCR8 monoclonal antibody	Phase I, +/- zemberelimab (anti-PD-1), in patients with advanced solid tumours	NCT05007782
LM-108	LaNova Medicines	Fc-optimised anti-CCR8 monoclonal antibody	Phase I/II, +/- toripalimab (anti-PD-1), in patients with advanced solid tumours	NCT05518045
AMG-355	Amgen	Anti-CCR8 monoclonal antibody	Phase I, +/- pembrolizumab (anti-PD-1), in patients with advanced solid tumours	NCT06131398
S-531011	Shionogi	Anti-CCR8 monoclonal antibody	Phase I/II, +/- pembrolizumab (anti-PD-1), in patients with advanced solid tumours	NCT05101070
BGB-3055	BeiGene	Anti-CCR8 monoclonal antibody	Phase I, +/- tislelizumab (anti-PD-1), in patients with advanced or metastatic solid tumours	NCT05935098
Adenosinergic Pathway				
TTX-030	Trishula Therapeutics/AbbVie	Anti-CD39 monoclonal antibody	Phase II, + chemotherapy +/- budigalimab (anti-PD-1), in patients with metastatic PDAC	NCT06119217
ES002023	Elpiscience Biopharma	Anti-CD39 monoclonal antibody	Phase I in patients with advanced solid tumours, including PDAC	NCT05075564
PUR001	Purinomia Biotech	Anti-CD39 monoclonal antibody	Phase I in patients with advanced solid tumours	NCT05234853
JS019	Shanghai Junshi Biosciences	Anti-CD39 monoclonal antibody	Phase I in patients with advanced solid tumours	NCT05508373
AB598	Arcus Biosciences	Anti-CD39 monoclonal antibody	Phase I, +/- zimberelimab (anti-PD-1) +/- chemotherapy, in patients with advanced solid tumours	NCT05891171
ES014	Elpiscience Biopharma	Anti-CD39-TGFb bispecific antibody	Phase I in patients with advanced or metastatic solid tumours	NCT05717348
Oleclumab (MEDI9447)	AstraZeneca	Anti-CD73 monoclonal antibody	Phase II, in combination with chemotherapy and durvalumab (anti-PD-L1), in patients with resectable/borderline resectable PDAC	NCT04940286
Mupadolimab (CPI-006)	Corvus Pharmaceuticals	Anti-CD73 monoclonal antibody	Phase Ib, +/- ciforadenant (selective A2aR antagonist) +/- pembrolizumab (anti-PD-1), in patients with advanced solid tumours including PDAC	NCT03454451
PT199	Phanes Therapeutics	Anti-CD73 monoclonal antibody	Phase I, +/- anti-PD-1 immunotherapy, in patients with advanced solid tumours including PDAC	NCT05431270
IPH5301	Innate Pharma	Anti-CD73 monoclonal antibody	Phase I, +/- trastuzumab (anti-HER2^†^) and paclitaxel, in patients with advanced solid tumours including metastatic PDAC	NCT05143970
HB0045	Shanghai Huaota Pharmaceuticals	Anti-CD73 monoclonal antibody	Phase I/II in patients with advanced solid tumours including PDAC	NCT06056323
INCA00186	Incyte Corporation	Anti-CD73 monoclonal antibody	Phase I, +/- INCB106385 (dual A2aR/A2bR antagonist) +/- retifanlimab (anti-PD-1), in patients with advanced solid tumours	NCT04989387
SYM024	Symphogen	Anti-CD73 monoclonal antibody	Phase I, +/- Sym021 (anti-PD-1), in patients with advanced solid tumours	NCT04672434
Uliledlimab (TJ004309)	I-Mab	Anti-CD73 monoclonal antibody	Phase I, in combination with toripalimab (anti-PD-1), in patients with advanced solid tumours	NCT04322006
JAB-BX102	Jacobio Pharmaceuticals	Anti-CD73 monoclonal antibody	Phase I, +/- pembrolizumab (anti-PD-1), in patients with advanced solid tumours	NCT05174585
Drebuxelimab (AK119)	Akeso	Anti-CD73 monoclonal antibody	Phase I, in combination with AK104 (anti-CTLA4-PD1 bispecific) or AK112 (anti-VEGF-PD1 bispecific^‡^), in patients with advanced solid tumours	NCT05559541, NCT05689853
PM1015	Biotheus	Anti-CD73 monoclonal antibody	Phase I in patients with advanced solid tumours	NCT05950815
Quemliclustat (AB680)	Arcus Biosciences/Gilead Sciences	Small-molecule, selective CD73 antagonist	Phase I, in combination with nab-paclitaxel and gemcitabine +/- zimberelimab (anti-PD-1), in patients with advanced PDAC	NCT04104672
ATG-037	Antengene Therapeutics	Small-molecule, selective CD73 antagonist	Phase I, +/- pembrolizumab (anti-PD-1), in patients with locally advanced or metastatic solid tumours	NCT05205109
Dalutrafusp alfa (AGEN1423)	Agenus Inc.	Anti-CD73-TGFb bispecific antibody	Phase II, in combination with balstilimab (anti-PD-1) +/- chemotherapy, in patients with advanced PDAC	NCT05632328
Ciforadenant (CPI-444)	Corvus Pharmaceuticals/Vernalis	Small-molecule, selective A2aR antagonist	Phase Ib, in combination with mupadolimab (anti-CD73), in patients with advanced solid tumours including PDAC	NCT03454451
Inupadenant (EOS-850)	iTeos Therapeutics	Small-molecule, selective A2aR antagonist	Phase I in patients with advanced solid tumours	NCT05060432
TT-10 (PORT-6)	Portage Biotech	Small-molecule, selective A2aR antagonist	Phase I in patients with advanced solid tumours	NCT04969315
ILB-2109	Innolake Biopharm	Small-molecule, selective A2aR antagonist	Phase I in patients with locally advanced or metastatic solid tumours	NCT05278546
Etrumadenant (AB928)	Arcus Biosciences	Small-molecule, dual A2aR/A2bR antagonist	Phase II, in combination with chemotherapy and atezolizumab (anti-PD-L1), in patients with metastatic PDAC	NCT03193190
TT-4 (PORT-7)	Portage Biotech	Small-molecule, selective A2bR antagonist	Phase I/II in patients with advanced solid tumours including PDAC	NCT04976660

* poliovirus receptor-related immunoglobulin domain-containing; ^†^ human epidermal growth factor receptor 2; ^‡^ vascular endothelial growth factor.

All information was obtained from the NIH clinical trials database (https://clinicaltrials.gov) or from the publicised development pipelines of pharmaceutical companies. The rows highlighted blue denote drugs that are under evaluation in clinical trials that include PDAC patients.

### 4.1 Re-engineering next-generation ICIs

Allison and colleagues originally attributed the anti-tumour activity of anti-CTLA-4 monoclonal antibodies (mAbs) to the reinvigoration of dysfunctional T_eff_ cells (56). However, accumulating evidence suggests that anti-CTLA-4 mAbs can preferentially deplete CTLA-4^hi^ T_regs_ *in vivo* by antibody-dependent cellular cytotoxicity (ADCC) (57–60). Thus, in spite of the failure of prior clinical trials (4, 61), this novel mechanistic insight provides a rationale for the continued development of anti-CTLA-4 mAbs to treat PDAC. Clearly, however, this will necessitate re-engineering of existing anti-CTLA-4 mAbs; specifically, the fragment crystallisable (F_c_) domain to enhance affinity for activatory F_c_γ receptors and decrease affinity for inhibitory receptors, thereby promoting ADCC. This approach can be optimised with consideration of the relative abundance and distribution of specific F_c_γRs on local effector cells; indeed, the engineering of anti-CTLA-4 mAbs in this manner has been shown to increase therapeutic activity in tumour-bearing mice (59, 62). Therefore, it is important that studies have identified intratumoral populations of F_c_γRIIIA (CD16)-expressing natural killer and myeloid cells in human PDAC (14–16). Moreover, Agenus recently initiated a phase I/II trial to investigate botensilimab – an F_c_-engineered anti-CTLA-4 mAb with enhanced affinity for F_c_γRIIIA – in metastatic PDAC patients (NCT05630183).

Further testament to the widespread interest in strategies to selectively deplete intratumoral T_regs_, there is renewed attention on the development of anti-CD25 mAbs. For example, Solomon et al. developed an anti-CD25 mAb (RG6292) that selectively depletes CD25^hi^ T_regs_, whilst preserving CD25-STAT5 signalling required for T_eff_ cell-mediated anti-tumour immunity (63). Indeed, a phase I trial of RG6292, conducted in patients with advanced/metastatic solid tumours, indicated a manageable safety profile and preliminary anti-tumour activity (64). However, multi-omic analysis of patient-derived tumour samples obtained during treatment with RG6292 is required to confirm this proposed mechanism of action *in vivo*.

### 4.2 Exploiting novel immune checkpoints

Since the discovery of CTLA-4 and PD-1, studies have identified a plethora of immune checkpoints – both inhibitory (e.g., TIGIT, LAG-3, TIM-3) and co-stimulatory (e.g., ICOS, OX40, GITR, 4–1BB) – that might be exploited therapeutically to augment anti-tumour immunity. In PDAC, TIGIT and ICOS are expressed at high levels on intratumoral eT_regs_ (14, 15). TIGIT is also expressed, albeit at lower levels, by dysfunctional T_eff_ cells, whereas ICOS is induced upon the activation of intratumoral T_eff_ cells (14, 15, 27). Therefore, anti-TIGIT and agonistic ICOS mAbs might have a dual mechanism of action: the re-invigoration of dysfunctional T_eff_ cells and selective depletion of activated T_regs_ (65). However, achieving the optimal balance between these mechanisms will require F_c_ engineering to effectively engage specific F_c_ receptors (66).

Tiragolumab (IgG1*κ* anti-TIGIT) has demonstrated tolerability and preliminary anti-tumour activity in patients with advanced solid tumours (67, 68). Consequently, two early-stage trials are investigating anti-TIGIT mAbs, incorporated into combinatorial regimens, for the treatment of metastatic PDAC (NCT03193190, NCT05419479). By contrast, a phase I/II trial, investigating vopratelimab (IgG1κ agonistic ICOS) for the treatment of advanced solid tumours, including three PDAC patients, reported limited efficacy (69). However, on-treatment emergence of ICOS^hi^ CD4^+^ T_eff_ cells was associated with therapeutic responses, suggesting that vopratelimab might indeed re-invigorate dysfunctional T_eff_ cells in patients through ICOS activation. More generally, this illustrates that multi-omic analyses of on-treatment patient-derived samples during clinical trials may further advance our understanding of the PDAC immune landscape.

### 4.3 De-stabilising activated T_regs_

The development of strategies for selectively drugging T_regs_ has been the subject of considerable research. One potential target is Helios; in PDAC patients, Helios^+^ T_regs_ are significantly enriched in the TME (70). Moreover, T_reg_-intrinsic deletion of Helios has been shown to enhance anti-tumour immunity in tumour-bearing mice (71). Interestingly, Helios-deficient T_regs_ acquire a T_eff_ phenotype including the production of pro-inflammatory cytokines (e.g., IFN-γ), which is attributed to downregulation of FOXP3 and de-repression of T_H_1/T_H_2 lineage determinants (43). In the absence of the stabilising influence of Helios, it appears that the inflammatory TME promotes the trans-differentiation of T_regs_ into activated T_eff_ cells. Intriguingly, this novel T_eff_ population is equipped with an inherently self-reactive TCR repertoire, which might be expected to direct a potent immune response against ‘quasi-self’ tumour antigens.

Transcription factors are traditionally considered difficult to drug. However, several recent studies have described small-molecules that selectively enhance the proteasomal degradation of Helios (72, 73). Future *in vivo* studies must determine whether these small-molecules can selectively destabilise activated intratumoral eT_regs_; one clinical trial is currently evaluating this approach in advanced solid tumours (NCT03891953).

### 4.4 Targeting chemokine receptors

The origin of intratumoral FOXP3^+^ T_regs_ is unclear – they may differentiate locally from T_eff_ cells or be recruited from the circulation. For the latter, targeting chemokine signalling axes (e.g., CCL2-CCR4; CCL5-CCR5) that can recruit T_regs_ into the PDAC TME is of interest. However, this strategy has proved disappointing thus far; clinical trials investigating mogamulizumab (IgG1 anti-CCR4) reported off-target depletion of T_H_2/T_H_17 cells, reflecting heterogeneous expression of CCR4 (74, 75).

It is notable, therefore, that intratumoral eT_regs_ uniquely express CCR8 (76). However, functional blockade of CCR8 does not affect T_reg_ recruitment; they acquire CCR8 expression in the TME, perhaps suggesting that this axis mediates retention of intratumoral T_regs_ (77). Nevertheless, CCR8 constitutes a target for the selective depletion of intratumoral eT_regs_ in PDAC. Pre-clinical studies have demonstrated that anti-CCR8 mAbs profoundly suppress tumour growth in tumour-bearing mice (76, 78). Further, this response coincided with the expansion of intratumoral CD4^+^ T_eff_ cells and the preservation of systemic T_reg_ populations, which may mitigate the risk of autoimmune-related adverse events. Currently, eight early-stage trials are investigating anti-CCR8 mAbs for the treatment of advanced solid tumours (NCT04895709, NCT06131398, NCT05635643, NCT05537740, NCT05007782, NCT05518045, NCT05101070, NCT05935098).

### 4.5 Combatting immunosuppressive adenosine

Apoptotic T_regs_ convert ATP to adenosine, an immunosuppressive metabolite, via ectoenzymes that remain catalytically active after cell-death. This raises the paradoxical possibility that the therapeutic depletion of T_regs_ might not limit T_reg_-cell-mediated immunosuppression. This discovery provided impetus to the development of immunotherapies that target the adenosinergic pathway: CD39, CD73, and the A_2A_/A_2B_ receptors. It is hoped that these therapies will synergise with T_reg_-targeted approaches, or other immunotherapeutic modalities, to induce potent anti-tumour immunity. To date, however, attempts to target this pathway with anti-CD73 mAbs have demonstrated no clinical benefit for PDAC patients; a phase-II trial investigating the combination of anti-CD73, anti-PD-L1, and chemotherapy revealed comparable efficacy to chemotherapy alone (79).

## 5 Conclusions and future perspectives

The manipulation of intratumoral T_regs_ may prove a valuable addition to our currently limited armamentarium for the treatment of PDAC. This therapeutic strategy has the potential to re-invigorate anti-tumour immunity by reprogramming the immunosuppressive milieu that is first established in pre-malignant lesions. This notion is supported by promising early-stage clinical trials of T_reg_-targeted immunotherapies (68, 80). Moreover, data from trials investigating anti-CCR8 mAbs and selective Helios degraders, strategies to selectively target intratumoral effector T_regs_, are eagerly awaited.

There are several outstanding questions, however, which threaten to hinder the effective therapeutic manipulation of intratumoral T_regs_:

1. Given that intratumoral T_regs_ are present from early carcinogenesis to the development of metastatic disease, are T_reg_-targeted therapies effective in cohorts of patients from the full spectrum of the natural history of PDAC?
2. With novel T_reg_-targeted interventions, is there on-treatment emergence of immunosuppressive FOXP3^-^ T_reg_-like cells (e.g., T_r_1 cells) or other complementary immunosuppressive mechanisms?
3. How can we prevent immune-related adverse events, which so often necessitate treatment discontinuation, when targeting T_regs_ for the treatment of PDAC?
4. To what extent do T_reg_-targeted therapies synergise with anti-cancer agents from our existing repertoire, including immunotherapies and conventional chemotherapies?

Importantly, with preliminary clinical evidence for the efficacy of T_reg_-targeted therapies, there is a compelling argument for the allocation of resources to resolve these outstanding questions.

## Author contributions

SS: Writing – original draft, Writing – review & editing. HS: Writing – original draft, Writing – review & editing. EA-B: Writing – original draft, Writing – review & editing. EA: Writing – original draft, Writing – review & editing.

## References

[1] Hanahan D Weinberg RA. Hallmarks of cancer: the next generation. Cell. (2011) 144:646–74. doi: 10.1016/j.cell.2011.02.013 21376230

[2] Busch W. Aus der Sitzung der medicinischen Section vom 13 November 1867. Berl Klin Wochenschr. (1868) 5:137.

[3] SChadendorf D Hodi FS Robert C Weber JS Margolin K Hamid O. Pooled analysis of long-term survival data from phase II and phase III trials of ipilimumab in unresectable or metastatic melanoma. J Clin Oncol. (2015) 33:1889–94. doi: 10.1200/JCO.2014.56.2736 PMC5089162 25667295

[4] O’Reilly EM Oh D-Y Dhani N Renouf DJ Lee MA Sun W. Durvalumab with or without tremelimumab for patients with metastatic pancreatic ductal adenocarcinoma: A phase 2 randomized clinical trial. JAMA Oncol. (2010) 5:1431–8. doi: 10.1001/jamaoncol.2019.1588 PMC6647002 31318392

[5] Mizrahi JD Surana R Valle JW Shroff RT. Pancreatic cancer. Lancet. (2020) 395:2008–20. doi: 10.1016/S0140-6736(20)30974-0 32593337

[6] International Agency for Research on Cancer. World Cancer Report 2020. Lyon, France: IARC Publications (2020). 367 p.

[7] Bockorny B Macarulla T Semenisty V Borazanci E Feliu J Ponz-Sarvise M. Motixafortide and pembrolizumab combined to nanoliposomal irinotecan, fluorouracil, and folinic acid in metastatic pancreatic cancer: the COMBAT/KEYNOTE-202 trial. Clin Cancer Res. (2021) 27:5020–7. doi: 10.1158/1078-0432.CCR-21-0929 34253578

[8] Bockorny B Semenisty V Macarulla T Borazanci E Wolpin BM Stemmer SM. BL-8040, a CXCR4 antagonist, in combination with pembrolizumab and chemotherapy for pancreatic cancer: the COMBAT trial. Nat Med. (2020) 26:878–85. doi: 10.1038/s41591-020-0880-x 32451495

[9] Hiraoka N Onozato K Kosuge T Hirohashi S. Prevalence of FOXP3+ Regulatory T cells increases during the progression of pancreatic ductal adenocarcinoma and its premalignant lesions. Clin Cancer Res. (2006) 12:5423–34. doi: 10.1158/1078-0432.CCR-06-0369 17000676

[10] Tumeh PC Harview CL Yearley JH Shintaku IP Taylor EJM Robert L. PD-1 blockade induces responses by inhibiting adaptive immune resistance. Nature. (2014) 515:568–71. doi: 10.1038/nature13954 PMC4246418 25428505

[11] Alspach E Lussier DM Miceli AP Kizhvatov I DuPage M Luoma AM. MHC-II neoantigens shape tumour immunity and response to immunotherapy. Nature. (2019) 574:696–701. doi: 10.1038/s41586-019-1671-8 31645760 PMC6858572

[12] Hingorani SR Petricoin EF III Maitra A Rajapakse V King C Jacobetz MA. Preinvasive and invasive ductal pancreatic cancer and its early detection in the mouse. Cancer Cell. (2003) 4:437–50. doi: 10.1016/S1535-6108(03)00309-X 14706336

[13] Hingorani SR Wang L Multani AS Combs C Deramaudt TB Hruban RH. Trp53R172H and KrasG12D cooperate to promote chromosomal instability and widely metastatic pancreatic ductal adenocarcinoma in mice. Cancer Cell. (2005) 7:469–83. doi: 10.1016/j.ccr.2005.04.023 15894267

[14] Steele NG Carpenter ES Kemp SB Sirihorachai VR The S Delrosario L. Multimodal mapping of the tumor and peripheral blood immune landscape in human pancreatic cancer. Nat Cancer. (2020) 1:1097–112. doi: 10.1038/s43018-020-00121-4 PMC8294470 34296197

[15] Sivakumar S Abu-Shah E Ahern DJ Arbe-Barnes EH Jainarayanan AK Mangal N. Activated regulatory T-cells, dysfunctional and senescent t-cells hinder the immunity in pancreatic cancer. Cancers (Basel). (2021) 13:1776. doi: 10.3390/cancers13081776 33917832 PMC8068251

[16] Sivakumar S Jainarayanan A Arbe-Barnes E Sharma PK Leathlobhair MN Amin S. Single-cell immune multi-omics and repertoire analyses in pancreatic ductal adenocarcinoma reveal differential immunosuppressive mechanisms within different tumour microenvironments. bioRxiv. (2023) 08.31.555730. doi: 10.1101/2023.08.31.555730

[17] Chen P-L Roh W Reuben A Cooper ZA Spencer CN Prieto PA. Analysis of immune signatures in longitudinal tumor samples yields insight into biomarkers of response and mechanisms of resistance to immune checkpoint blockade. Cancer Discovery. (2016) 6:827–37. doi: 10.1158/2159-8290.CD-15-1545 PMC5082984 27301722

[18] Spranger S Koblish HK Horton B Scherle PA Newton R Gajewski TF. Mechanism of tumor rejection with doublets of CTLA-4, PD-1/PD-L1, or IDO blockade involves restored IL-2 production and proliferation of CD8+ T cells directly within the tumor microenvironment. J Immunother Cancer. (2014) 2:3. doi: 10.1186/2051-1426-2-3 24829760 PMC4019906

[19] Clark CE Hingorani SR Mick R Combs C Tuveson DA Vonderheide RH. Dynamics of the immune reaction to pancreatic cancer from inception to invasion. Cancer Res. (2007) 67:9518–27. doi: 10.1158/0008-5472.CAN-07-0175 17909062

[20] Bear AS Vonderheide RH O’Hara MH. Challenges and opportunities for pancreatic cancer immunotherapy. Cancer Cell. (2020) 38:788–802. doi: 10.1016/j.ccell.2020.08.004 32946773 PMC7738380

[21] Carstens JL Correa de Sampaio P Yang D Barua S Wang H Rao A. Spatial computation of intratumoral T cells correlates with survival of patients with pancreatic cancer. Nat Commun. (2017) 8:15095. doi: 10.1038/ncomms15095 28447602 PMC5414182

[22] Ene–Obong A Clear AJ Watt J Wang J Fatah R Riches JC. Activated pancreatic stellate cells sequester CD8+ T cells to reduce their infiltration of the juxtatumoral compartment of pancreatic ductal adenocarcinoma. Gastroenterology. (2013) 145:1121–32. doi: 10.1053/j.gastro.2013.07.025 PMC3896919 23891972

[23] Balli D Rech AJ Stanger BZ Vonderheide RH. Immune cytolytic activity stratifies molecular subsets of human pancreatic cancer. Clin Cancer Res. (2017) 23:3129–38. doi: 10.1158/1078-0432.CCR-16-2128 PMC12164831 28007776

[24] Balachandran VP Łuksza M Zhao JN Makarov V Moral JA Remark R. Identification of unique neoantigen qualities in long-term survivors of pancreatic cancer. Nature. (2017) 551:512–6. doi: 10.1038/nature24462 PMC6145146 29132146

[25] Jainarayanan A Mouroug-Anand N Arbe-Barnes EH Bush AJ Bashford-Rogers R Frampton A. Pseudotime dynamics of T cells in pancreatic ductal adenocarcinoma inform distinct functional states within the regulatory and cytotoxic T cells. iScience. (2023) 26:106324. doi: 10.1016/j.isci.2023.106324 36968070 PMC10034436

[26] de Santiago I Yau C Heij L Middleton MR Markowetz F Grabsch HI. Immunophenotypes of pancreatic ductal adenocarcinoma: Meta-analysis of transcriptional subtypes. Int J Cancer. (2019) 145:1125–37. doi: 10.1002/ijc.32186 PMC6767191 30720864

[27] Freed-Pastor WA Lambert LJ Ely ZA Pattada NB Bhutkar A Eng G. The CD155/TIGIT axis promotes and maintains immune evasion in neoantigen-expressing pancreatic cancer. Cancer Cell. (2021) 39:1342–1360.e14. doi: 10.1016/j.ccell.2021.07.007 34358448 PMC8511341

[28] Lupinacci RM Goloudina A Buhard O Bachet J-B Maréchal R Demetter P. Prevalence of microsatellite instability in intraductal papillary mucinous neoplasms of the pancreas. Gastroenterology. (2018) 154:1061–5. doi: 10.1053/j.gastro.2017.11.009 29158190

[29] McGranahan N Furness AJS Rosenthal R Ramskov S Lyngaa R Saini SK. Clonal neoantigens elicit T cell immunoreactivity and sensitivity to immune checkpoint blockade. Science. (2016) 351:1463–9. doi: 10.1126/science.aaf1490 PMC4984254 26940869

[30] Li B Li T Pignon J-C Wang B Wang J Shukla SA. Landscape of tumor-infiltrating T cell repertoire of human cancers. Nat Genet. (2016) 48:725–32. doi: 10.1038/ng.3581 PMC5298896 27240091

[31] Le DT Durham JN Smith KN Wang H Bartlett BR Aulakh LK. Mismatch repair deficiency predicts response of solid tumors to PD-1 blockade. Science. (2017) 357:409–13. doi: 10.1126/science.aan6733 PMC5576142 28596308

[32] Gros A Tran E Parkhurst MR Ilyas S Pasetto A Groh EM. Recognition of human gastrointestinal cancer neoantigens by circulating PD-1+ lymphocytes. J Clin Invest. (2019) 129:4992–5004. doi: 10.1172/JCI127967 31609250 PMC6819109

[33] Parkhurst MR Robbins PF Tran E Prickett TD Gartner JJ Jia L. Unique neoantigens arise from somatic mutations in patients with gastrointestinal cancers. Cancer Discovery. (2019) 9:1022–35. doi: 10.1158/2159-8290.CD-18-1494 PMC7138461 31164343

[34] Huber M Brehm CU Gress TM Buchholz M Alhamwe BA von Strandmann EP. The immune microenvironment in pancreatic cancer. Int J Mol Sci. (2020) 21:7307. doi: 10.3390/ijms21197307 33022971 PMC7583843

[35] Huang H Wang Z Zhang Y Pradhan RN Ganguly D Chandra R. Mesothelial cell-derived antigen-presenting cancer-associated fibroblasts induce expansion of regulatory T cells in pancreatic cancer. Cancer Cell. (2022) 40:656–73. doi: 10.1016/j.ccell.2022.04.011 PMC9197998 35523176

[36] Leinwand J Miller G. Regulation and modulation of antitumor immunity in pancreatic cancer. Nat Immunol. (2020) 21:1152–9. doi: 10.1038/s41590-020-0761-y 32807942

[37] Padrón LJ Maurer DM O’Hara MH O’Reilly EM Wolff RA Wainberg ZA. Sotigalimab and/or nivolumab with chemotherapy in first-line metastatic pancreatic cancer: clinical and immunologic analyses from the randomized phase 2 PRINCE trial. Nat Med. (2022) 28:1167–77. doi: 10.1038/s41591-022-01829-9 PMC9205784 35662283

[38] Kim EJ Sahai V Abel EV Griffith KA Greenson JK Takebe N. Pilot clinical trial of hedgehog pathway inhibitor GDC-0449 (vismodegib) in combination with gemcitabine in patients with metastatic pancreatic adenocarcinoma. Clin Cancer Res. (2014) 20:5937–45. doi: 10.1158/1078-0432.CCR-14-1269 PMC4254161 25278454

[39] Benson AB Wainberg ZA Hecht JR Vyushkov D Dong H Bendell J. A phase II randomized, double-blind, placebo-controlled study of simtuzumab or placebo in combination with gemcitabine for the first-line treatment of pancreatic adenocarcinoma. Oncologist. (2017) 22:241–e15. doi: 10.1634/theoncologist.2017-0024 28246206 PMC5344644

[40] Zhen DB Whittle M Ritch PS Hochster HS Coveler AL George B. Phase II study of PEGPH20 plus pembrolizumab for patients (pts) with hyaluronan (HA)-high refractory metastatic pancreatic adenocarcinoma (mPC). J Clin Oncol. (2022) 40:576. doi: 10.1200/JCO.2022.40.4-suppl.576 34985931

[41] Jang J-E Hajdu CH Liot C Miller G Dustin ML Bar-Sagi D. Crosstalk between regulatory T cells and tumor-associated dendritic cells negates anti-tumor immunity in pancreatic cancer. Cell Rep. (2017) 20:558–71. doi: 10.1016/j.celrep.2017.06.062 PMC5649374 28723561

[42] Zhang Y Lazarus J Steele NG Yan W Lee H-J Nwosu ZC. Regulatory T-cell depletion alters the tumor microenvironment and accelerates pancreatic carcinogenesis. Cancer Discovery. (2020) 10:422–39. doi: 10.1158/2159-8290.CD-19-0958 PMC7224338 31911451

[43] Yates K Bi K Haining WN Cantor H Kim H-J. Comparative transcriptome analysis reveals distinct genetic modules associated with Helios expression in intratumoral regulatory T cells. Proc Natl Acad Sci USA. (2018) 115:2162–7. doi: 10.1073/pnas.1720447115 PMC5834721 29440380

[44] Kim H-J Barnitz RA Kreslavsky T Brown FD Moffett H Lemieux ME. Stable inhibitory activity of regulatory T cells requires the transcription factor Helios. Science. (2015) 350:334–9. doi: 10.1126/science.aad0616 PMC4627635 26472910

[45] Sharabi A Tsokos MG Ding Y Malek TR Klatzmann D Tsokos GC. Regulatory T cells in the treatment of disease. Nat Rev Drug Discovery. (2018) 17:823–44. doi: 10.1038/nrd.2018.148 30310234

[46] Chellappa S Hugenschmidt H Hagness M Line PD Labori KJ Wiedswang G. Regulatory T cells that co-express RORγt and FOXP3 are pro-inflammatory and immunosuppressive and expand in human pancreatic cancer. Oncoimmunology. (2016) 5:e1102828. doi: 10.1080/2162402X.2015.1102828 27141387 PMC4839385

[47] McAllister F Bailey JM Alsina J Nirschl CJ Sharma R Fan H. Oncogenic kras activates a hematopoietic-to-epithelial IL-17 signaling axis in preinvasive pancreatic neoplasia. Cancer Cell. (2014) 25:621–37. doi: 10.1016/j.ccr.2014.03.014 PMC4072043 24823639

[48] Barilla RM Diskin B Caso RC Lee KB Mohan N Buttar C. Specialized dendritic cells induce tumor-promoting IL-10+IL-17+ FoxP3neg regulatory CD4+ T cells in pancreatic carcinoma. Nat Commun. (2019) 10:1424. doi: 10.1038/s41467-019-09416-2 30926808 PMC6441038

[49] Whiteside SK Grant FM Alvisi G Clarke J Tang L Imianowski CJ. Acquisition of suppressive function by conventional T cells limits antitumor immunity upon Treg depletion. Sci Immunol. (2023) 8:eabo5558. doi: 10.1126/sciimmunol.abo5558 38100544 PMC7615475

[50] Maj T Wang W Crespo J Zhang H Wang W Wei S. Oxidative stress controls regulatory T cell apoptosis and suppressor activity and PD-L1-blockade resistance in tumor. Nat Immunol. (2017) 18:1332–41. doi: 10.1038/ni.3868 PMC5770150 29083399

[51] Deaglio S Dwyer KM Gao W Friedman D Usheva A Erat A. Adenosine generation catalyzed by CD39 and CD73 expressed on regulatory T cells mediates immune suppression. J Exp Med. (2007) 204:1257–65. doi: 10.1084/jem.20062512 PMC2118603 17502665

[52] Loi S Pommey S Haibe-Kains B Beavis PA Darcy PK Smyth MJ. CD73 promotes anthracycline resistance and poor prognosis in triple negative breast cancer. Proc Natl Acad Sci USA. (2013) 110:11091–6. doi: 10.1073/pnas.1222251110 PMC3704029 23776241

[53] Wu X-R He X-S Chen Y-F Yuan R-X Zeng Y Lian L. High expression of CD73 as a poor prognostic biomarker in human colorectal cancer. J Surg Oncol. (2012) 106:130–7. doi: 10.1002/jso.23056 22287455

[54] Onizuka S Tawara I Shimizu J Sakaguchi S Fujita T Nakayama E. Tumor rejection by in vivo administration of anti-CD25 (Interleukin-2 receptor α) monoclonal antibody. Cancer Res. (1999) 59:3128–33. 10397255

[55] Litzinger MT Fernando R Curiel TJ Grosenbach DW Schlom J Palena C. IL-2 immunotoxin denileukin diftitox reduces regulatory T cells and enhances vaccine-mediated T-cell immunity. Blood. (2007) 110:3192–201. doi: 10.1182/blood-2007-06-094615 PMC2200901 17616639

[56] Krummel MF Allison JP. CD28 and CTLA-4 have opposing effects on the response of T cells to stimulation. J Exp Med. (1995) 182:459–65. doi: 10.1084/jem.182.2.459 PMC2192127 7543139

[57] Simpson TR Li F Montalvo-Ortiz W Sepulveda MA Bergerhoff K Arce F. Fc-dependent depletion of tumor-infiltrating regulatory T cells co-defines the efficacy of anti–CTLA-4 therapy against melanoma. J Exp Med. (2013) 210:1695–710. doi: 10.1084/jem.20130579 PMC3754863 23897981

[58] Selby MJ Engelhardt JJ Quigley M Henning KA Chen T Srinivasan M. Anti-CTLA-4 antibodies of igG2a isotype enhance antitumor activity through reduction of intratumoral regulatory T cells. Cancer Immunol Res. (2013) 1:32–42. doi: 10.1158/2326-6066.CIR-13-0013 24777248

[59] Arce Vargas F Furness AJS Litchfield K Joshi K Rosenthal R Ghorani E. Fc effector function contributes to the activity of human anti-CTLA-4 antibodies. Cancer Cell. (2018) 33:649–63. doi: 10.1016/j.ccell.2018.02.010 PMC5904288 29576375

[60] Romano E Kusio-Kobialka M Foukas PG Baumgaertner P Meyer C Ballabeni P. Ipilimumab-dependent cell-mediated cytotoxicity of regulatory T cells ex vivo by nonclassical monocytes in melanoma patients. Proc Natl Acad Sci USA. (2015) 112:6140–5. doi: 10.1073/pnas.1417320112 PMC4434760 25918390

[61] Royal RE Levy C Turner K Mathur A Hughes M Kammula US. Phase 2 trial of single agent ipilimumab (Anti-CTLA-4) for locally advanced or metastatic pancreatic adenocarcinoma. J Immunother. (2010) 33:828–33. doi: 10.1097/CJI.0b013e3181eec14c PMC7322622 20842054

[62] Knorr DA Blanchard L Leidner RS Jensen SM Meng R Jones A. FcγRIIB is an immune checkpoint limiting the activity of treg-targeting antibodies in the tumor microenvironment. Cancer Immunol Res. (2024) 12:322–33. doi: 10.1158/2326-6066.CIR-23-0389 PMC10911703 38147316

[63] Solomon I Amann M Goubier A Arce Vargas F Zervas D Qing C. CD25-Treg-depleting antibodies preserving IL-2 signaling on effector T cells enhance effector activation and antitumor immunity. Nat Cancer. (2020) 1:1153–66. doi: 10.1038/s43018-020-00133-0 PMC7116816 33644766

[64] Gambardella V Ong M Ruiz MER Machiels J-P de Sanmamed MF Tabernero JM. Abstract CT110: Safety and anti-tumor activity of a novel Treg depleter RG6292, as a single agent and in combination with atezolizumab in patients with solid tumors. Cancer Res. (2023) 83:CT110. doi: 10.1158/1538-7445.am2023-ct110 PMC11891644 39983024

[65] Sainson RCA Thotakura AK Kosmac M Borhis G Parveen N Kimber R. An antibody targeting ICOS increases intratumoral cytotoxic to regulatory T-cell ratio and induces tumor regression. Cancer Immunol Res. (2020) 8:1568–82. doi: 10.1158/2326-6066.CIR-20-0034 32999002

[66] Kraehenbuehl L Weng C-H Eghbali S Wolchok JD Merghoub T. Enhancing immunotherapy in cancer by targeting emerging immunomodulatory pathways. Nat Rev Clin Oncol. (2022) 19:37–50. doi: 10.1038/s41571-021-00552-7 34580473

[67] Bendell JC Bedard P Bang Y-J LoRusso P Hodi S Gordon M. Abstract CT302: Phase Ia/Ib dose-escalation study of the anti-TIGIT antibody tiragolumab as a single agent and in combination with atezolizumab in patients with advanced solid tumors. Cancer Res. (2020) 80:302. doi: 10.1158/1538-7445.AM2020-CT302

[68] Cho BC Abreu DR Hussein M Cobo M Patel AJ Secen N. Tiragolumab plus atezolizumab versus placebo plus atezolizumab as a first-line treatment for PD-L1-selected non-small-cell lung cancer (CITYSCAPE): primary and follow-up analyses of a randomised, double-blind, phase 2 study. Lancet Oncol. (2022) 23:781–92. doi: 10.1016/S1470-2045(22)00226-1 35576957

[69] Yap TA Gainor JF Callahan MK Falchook GS Pachynski RK LoRusso P. First-in-human phase I/II ICONIC trial of the ICOS agonist vopratelimab alone and with nivolumab: ICOS-high CD4 T-cell populations and predictors of response. Clin Cancer Res. (2022) 28:3695–708. doi: 10.1158/1078-0432.CCR-21-4256 PMC9433959 35511938

[70] Liu M-F Jin C Wu T Chen E-H Lu M Qin H-L. Helios serves as a suppression marker to reduce regulatory T cell function in pancreatic cancer patients. Immunol Res. (2021) 69:275–84. doi: 10.1007/s12026-021-09200-9 33959834

[71] Nakagawa H Sido JM Reyes EE Kiers V Cantor H Kim H-J. Instability of Helios-deficient Tregs is associated with conversion to a T-effector phenotype and enhanced antitumor immunity. Proc Natl Acad Sci USA. (2016) 113:6248–53. doi: 10.1073/pnas.1604765113 PMC4896716 27185917

[72] Wang ES Verano AL Nowak RP Yuan JC Donovan KA Eleuteri NA. Acute pharmacological degradation of Helios destabilizes regulatory T cells. Nat Chem Biol. (2021) 17:711–7. doi: 10.1038/s41589-021-00802-w PMC8162940 34035522

[73] Bonazzi S d’Hennezel E Beckwith REJ Xu L Fazal A Magracheva A. Discovery and characterization of a selective IKZF2 glue degrader for cancer immunotherapy. Cell Chem Biol. (2023) 30:235–247.e12. doi: 10.1016/j.chembiol.2023.02.005 36863346

[74] Kurose K Ohue Y Wada H Iida S Ishida T Kojima T. Phase ia study of foxP3+ CD4 treg depletion by infusion of a humanized anti-CCR4 antibody, KW-0761, in cancer patients. Clin Cancer Res. (2015) 21:4327–36. doi: 10.1158/1078-0432.CCR-15-0357 26429981

[75] Doi T Muro K Ishii H Kato T Tsushima T Takenoyama M. A phase I study of the anti-CC chemokine receptor 4 antibody, mogamulizumab, in combination with nivolumab in patients with advanced or metastatic solid tumors. Clin Cancer Res. (2019) 25:6614–22. doi: 10.1158/1078-0432.CCR-19-1090 31455681

[76] Kidani Y Nogami W Yasumizu Y Kawashima A Tanaka A Sonoda Y. CCR8-targeted specific depletion of clonally expanded Treg cells in tumor tissues evokes potent tumor immunity with long-lasting memory. Proc Natl Acad Sci USA. (2022) 119:e2114282119. doi: 10.1073/pnas.2114282119 35140181 PMC8851483

[77] Whiteside SK Grant FM Gyori DS Conti AG Imianowski CJ Kuo P. CCR8 marks highly suppressive Treg cells within tumours but is dispensable for their accumulation and suppressive function. Immunology. (2021) 163:512–20. doi: 10.1111/imm.13337 PMC8274197 33838058

[78] Campbell JR McDonald BR Mesko PB Siemers NO Singh PB Selby M. Fc-optimized anti-CCR8 antibody depletes regulatory T cells in human tumor models. Cancer Res. (2021) 81:2983–94. doi: 10.1158/0008-5472.CAN-20-3585 33757978

[79] Coveler AL Reilley M Zalupski M Macarulla T Fountzilas C Castanon Alvarez E. Safety and clinical activity of oleclumab (O) ± durvalumab (D) + chemotherapy (CT) in patients (pts) with metastatic pancreatic ductal adenocarcinoma (mPDAC): A phase 1b/2 randomized study. J Clin Oncol. (2023) 41:4136. doi: 10.1200/JCO.2023.41.16_suppl.4136

[80] Manji GA Wainberg ZA Krishnan K Giafis N Udyavar A Quah CS. ARC-8: Phase I/Ib study to evaluate safety and tolerability of AB680 + chemotherapy + zimberelimab (AB122) in patients with treatment-naive metastatic pancreatic adenocarcinoma (mPDAC). J Clin Oncol. (2021) 39:404. doi: 10.1200/JCO.2021.39.3_suppl.404

